# Effectiveness of combining microcurrent with resistance training in trained males

**DOI:** 10.1007/s00421-019-04243-1

**Published:** 2019-10-17

**Authors:** Fernando Naclerio, Marcos Seijo, Bettina Karsten, George Brooker, Leandro Carbone, Jack Thirkell, Eneko Larumbe-Zabala

**Affiliations:** 1 Department of Life and Sport Science, University of Greenwich, Avery Hill Campus, Sparrows Farm, Avery Hill Road, Eltham, SE9 2BT UK; 2 Department of Exercise and Sport Science, Lunex International University of Health, Exercise and Sports, Differdange, Luxemburg; 3 Department of Biological Sciences, Royal Holloway, University of London, London, UK; 4 Clinical Research Institute, Texas Tech University Health Sciences Center, Lubbock, TX USA

**Keywords:** Strength, Muscle thickness, DOMS, Hypertrophy, Non-invasive electrical microampere stimulus

## Abstract

**Introduction:**

Microcurrent has been used to promote tissue healing after injury or to hasten muscle remodeling post exercise post exercise.

**Purpose:**

To compare the effects of resistance training in combination with either, microcurrent or sham treatment, on-body composition and muscular architecture. Additionally, changes in performance and perceived delayed onset muscle soreness (DOMS) were determined.

**Methods:**

Eighteen males (25.7 ± 7.6 years) completed an 8-week resistance training program involving 3 workouts per week (24 total sessions) wearing a microcurrent (MIC, *n* = 9) or a sham (SH, *n* = 9) device for 3-h post-workout or in the morning during non-training days. Measurements were conducted at pre and post intervention.

**Results:**

Compared to baseline, both groups increased (*p* < 0.05) muscle thickness of the elbow flexors (MIC + 2.9 ± 1.4 mm; SH + 3.0 ± 2.4 mm), triceps brachialis (MIC + 4.3 ± 2.8 mm; SH + 2.7 ± 2.6 mm), vastus medialis (MIC + 1.5 ± 1.5 mm; SH + 0.9 ± 0.8 mm) and vastus lateralis (MIC + 6.8 ± 8.0 mm; SH + 3.2 ± 1.8 mm). Although both groups increased (*p* < 0.01) the pennation angle of vastus lateralis (MIC + 2.90° ± 0.95°; SH + 1.90° ± 1.35°, *p* < 0.01), the change measured in MIC was higher (*p* = 0.045) than that observed in SH. Furthermore, only MIC enlarged (*p* < 0.01) the pennation angle of brachialis (MIC + 1.93 ± 1.51). Both groups improved (*p* < 0.05) bench press strength and power but only MIC enhanced (*p* < 0.01) vertical jump height. At post intervention, only MIC decreased (*p* < 0.05) DOMS at 12-h, 24-h, and 48-h after performing an exercise-induced muscle soreness protocol.

**Conclusion:**

A 3-h daily use of microcurrent maximized muscular architectural changes and attenuated DOMS with no added significant benefits on body composition and performance.

## Introduction

Microcurrent-based treatments were proposed more than 30 years ago (McMakin 2004). This technology requires the use of an electrical device generating currents in the microampere (μA) range (1 μA equals 1/1000th of a milliamp). There is no physical sensation associated with the application of a microcurrent as the current intensity is not high enough to stimulate sensory nerve fibers (Mercola and Kirsch 1995). Some in vitro studies have revealed that the application of electric fields and currents similar to those generated within the human body can substantially change cell metabolism (Huckfeldt et al. 2007), optimizing tissue healing and injury repair (Ahmed et al. 2012) or promoting situations associated with high level of physiological stress as occurring during hard exercise sessions (Owens et al. 2018). The rationale behind the application of electrical currents is based on an increased ability of the cell to generate electric currents with biological effects across both cell and mitochondrial membranes (McCaig et al. 2005). Action potentials are generated by active transport of ions across the membranes, enabling the cell to work as a battery, in turn enhancing its efficiency to generate adenosine triphosphate (ATP) (Reid and Zhao 2014). In fact, the application of microcurrent has been associated with several health-related benefits such as (1) an increased number of mitochondria (Noites et al. 2015), (2) an improved ability to produce ATP (Noites et al. 2015), (3) a more efficient amino acid transport which promotes protein synthesis (Curtis et al. 2010), and satellite cell proliferation (Moon et al. 2018), (4) a faster regrowth of atrophied soleus muscle in mice (Ohno et al. 2013), and (5) the activation of hormone sensitive lipase, which can increase lipolysis from the internal and external adipose tissue (Noites et al. 2015). These proposed effects support the notion that combining microcurrent interventions with exercise might aid recovery but also it might elicit superior training outcomes. In this context, combining microcurrent with training could be an effective strategy for improving muscle function during exercise, attenuate muscle damage and optimize recovery (Kwon et al. 2017) by maximizing the skeletal muscle protein synthesis response (Ohno et al. 2019) and increasing the mitotic activity of satellite cells (Park et al. 2019). Recent studies in animals suggested positive effects of microcurrent to increase MM isoenzyme of creatine kinase, a marker of myogenic differentiation (Ohno et al. 2019) and to activate intracellular signalling pathways involved in the activation of the mechanistic target of rapamycin complex 1 (mTORC1) (Moon et al. 2018; Ohno et al. 2019). Furthermore, compared to sham treatment microcurrent therapy can also prevent muscle damage (Lambert et al. 2002; Kwon et al. 2017). Lambert et al. (2002) observed positive effects of a 96-h microcurrent protocol in the reduction of symptoms associated with muscle damage after performing 5 sets of 25 eccentric contractions of the elbow flexors at 80% of the maximal eccentric force. Results demonstrated a reduced muscle shortening and a delayed onset muscle soreness (DOMS). Similarly Curtis et al. (2010), reported a significant reduction of DOMS after performing 5 sets of 15 maximal voluntary leg curl eccentric contraction following the exposure of a 20 min microcurrent stimulation applied at an intensity of 200 μA and frequencies between 40 and 191 Hz in healthy adults. More recently Noites et al. (2015), reported promising results when combining a microcurrent treatment with endurance training, as it significantly reduced internal fat deposition when compared to performing exercise alone. Moreover, an acute enhancement effect on muscular function has also been reported in healthy elderly individuals after being exposed to a short-term 40 min microcurrent protocol (Kwon et al. 2017).

Even though the use of microcurrent has been empirically reported as a practical and effective method to augment training adaptation (Curtis et al. 2010), to the best of the authors’ knowledge there is a paucity of research aimed to verify the effects of microcurrent treatments on exercise adaptations and performance outcomes in athletes or regular fitness exercisers. The aim of this investigation, therefore, was to analyze the effects of adding a daily microcurrent treatment using a complex pulsed waveform with a fundamental frequency of 1.0309 kHz along with a variety of current intensities between 50 and 400 μA, to a regular resistance exercise program on training-induced outcomes in resistance-trained young male individuals. Given the potential benefits of microcurrent in promoting growth and remodeling in animal skeletal muscles (Ohno et al. 2013, 2019; Fujiya et al. 2015), the primary outcome measures were changes in body composition and muscle architecture. Due to its impact in limiting recovery of the muscular function following hard exercise protocols (Udani et al. 2009) secondary outcome measures included changes in performance and the perception of muscular soreness. Based on the available literature and compared to a sham condition, we hypothesized that the microcurrent treatment maximizes training outcomes and that it attenuates the perception of DOMS.

## Methods

### Experimental design

The study utilized a two parallel group randomized controlled trial design. Participants were randomly allocated into one of the two intervention groups: (1) microcurrent (MIC; *n* = 9) or (2) sham (SH; *n* = 9). Measures of body composition, muscular architecture (thickness and pennation angle), performance and muscle soreness were assessed before and after an 8-week intervention period. Following the initial assessment, participants were matched by body mass (BM) and maximal strength measured in the bench press (BP) exercise. The assignment of participants to treatments was performed by block randomization, using a block size of two, and in a double-blind fashion. Both groups performed an identical three-session per week resistance training routine. Participants received either a 3-h daily intervention to a microcurrent or sham exposure immediately post workout or during the morning on non-training days.

### Participants

To be eligible, participants had to be aged between 18 and 45 years, have at least 2 years of resistance training experience with a minimum training frequency of 2 days per week. Only resistance-trained individuals attending gyms or fitness centers who did not engage in sports competitions including bodybuilding, powerlifting or weightlifting, were considered. Participants also had to be free of (1) any existing or residual musculoskeletal injury within the last 3 months prior to the intervention (2) metabolic conditions (3) diseases (4) smoking (5) use of medications and (6) consuming nutritional supplements known to affect physical performance, muscle damage or recovery processes (e.g. creatine, isolate or hydrolysate protein extracts, amino acids, etc.) within 12 weeks prior to the start of the study. The study was approved by the institutional University Research Ethics Committee and all procedures were in accordance with the declaration of Helsinki. Prior to signing written informed consent, participants were fully informed about the nature and risks of the study. The project was registered as a clinical trial at the U.S. National Institutes of Health. https://www.clinicaltrials.gov (NCT03477747).

**Fig. 1:**
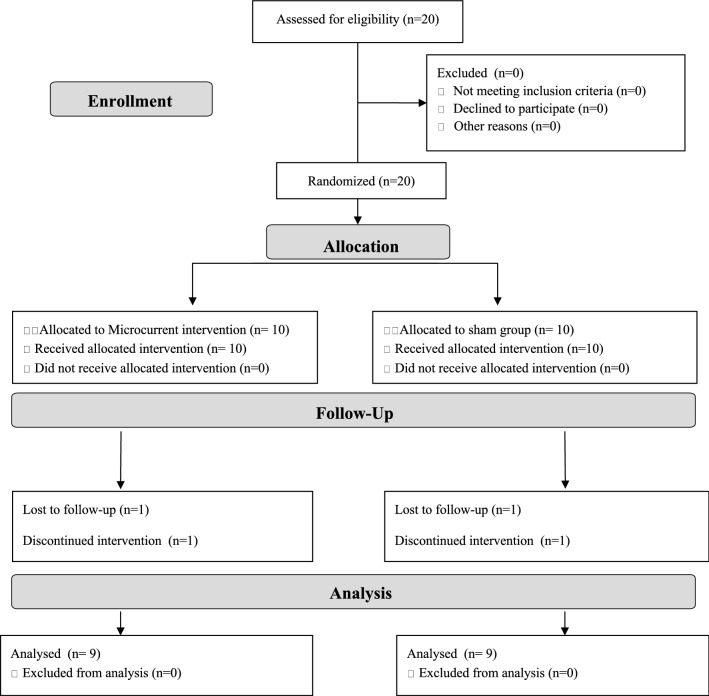
Flow diagram of participants throughout the course of the study

To determine the appropriate sample size, an interim analysis was performed once 12 participants (*n* = 6 per group) completed the study. Effect sizes were calculated using ANCOVA for muscle thickness outcome variables adjusted for their respective baseline levels. The interim analysis revealed large effect sizes for the main upper body (elbow flexors, *d* = 2.55) and lower body muscle thickness (vastus medialis, *d* = 1.50) variables. With a confidence level of 0.05 and power of 80%, it was determined that 18 participants (9 per group) would be necessary to achieve statistical significance for the difference between groups in the primary outcome measure (elbow flexors thickness and vastus medialis thickness). As summarized in Fig. 1, twenty participants were randomly allocated into one of the two intervention groups (MIC or SH). Eighteen of the twenty initially recruited participants completed all aspects of the intervention protocol and were considered for the final analysis.

Presented as mean ± standard deviation the final composition of the groups was as follows: MIC (*n* = 9): age: 26.1 ± 6.5 years; height: 178.1 ± 2.9 cm; BM: 87.9 ± 11.1 kg; 1RM BP: 100.6 ± 21.7 kg. SH (*n* = 9): age: 25.2 ± 8.5 years; height: 184.5 ± 5.6 cm; and BM: 89.5 ± 10.3 kg; 1RM BP: 96.67 ± 19.4 kg.

### Procedures

*Familiarization* Even though participants were experienced with resistance training, the study aimed to control learning effects by familiarization over a 1-week period involving three sessions. After that and during the first session, the training routine and intervention procedures were once more explained and demonstrated. To ensure that the intervention (workout and the use of the microcurrent or sham device) was conducted in accordance with the protocol, participants received a personalized follow-up during the 8 weeks of intervention.

*Assessments* Participants refrained from heavy exercise during 48-h prior to all assessments. Baseline values of all variables were tested within 1 day and in the following order: (1) body composition (2) muscular architecture (3) vertical jump (4) upper body BP strength (5) upper body BP power and (6) exercise-induced muscle soreness protocol (EIMS). A passive recovery period of 10 min was provided between each individual test.

*Body composition* The standard measurements were performed in accordance with the recommendations for anthropometric assessment (Ross and Marfell-Jones 1991). To eliminate inter-observer variability, only one investigator consistently performed all measurements. Height was measured in a stretched stature to the nearest 0.01 m using a wall-mounted stadiometer (Seca GmbH, Hamburg, Germany) and BM was corrected to the nearest 0.1 kg using a digital scale (Seca GmbH, Hamburg, Germany). Fat mass (FM) and fat-free mass (FFM) were estimated from whole body densitometry using air displacement via Bod Pod^®^ (Life Measurements, Concord, CA, USA) and following the manufacturer’s instructions as detailed elsewhere (Dempster and Aitkens 1995).

*Muscular architecture* A real-time B-mode ultrasound imaging system (Philips Affiniti 70 Ultrasound, Philips Corporation, USA) was used to measure changes in muscular architecture under static conditions. In accordance with the protocol described by Bradley and O’Donnell (2002) a trained researcher performed all measurements in a standardized manner. Using ultrasonography of the cross-sectional area and determined on the dominant side, the thickness of elbow flexors (EF), triceps brachii (TB), vastus medialis (VM) and vastus lateralis (VL), along with the fiber pennation angle of brachialis (BR) and VL were assessed.

**Fig. 2:**
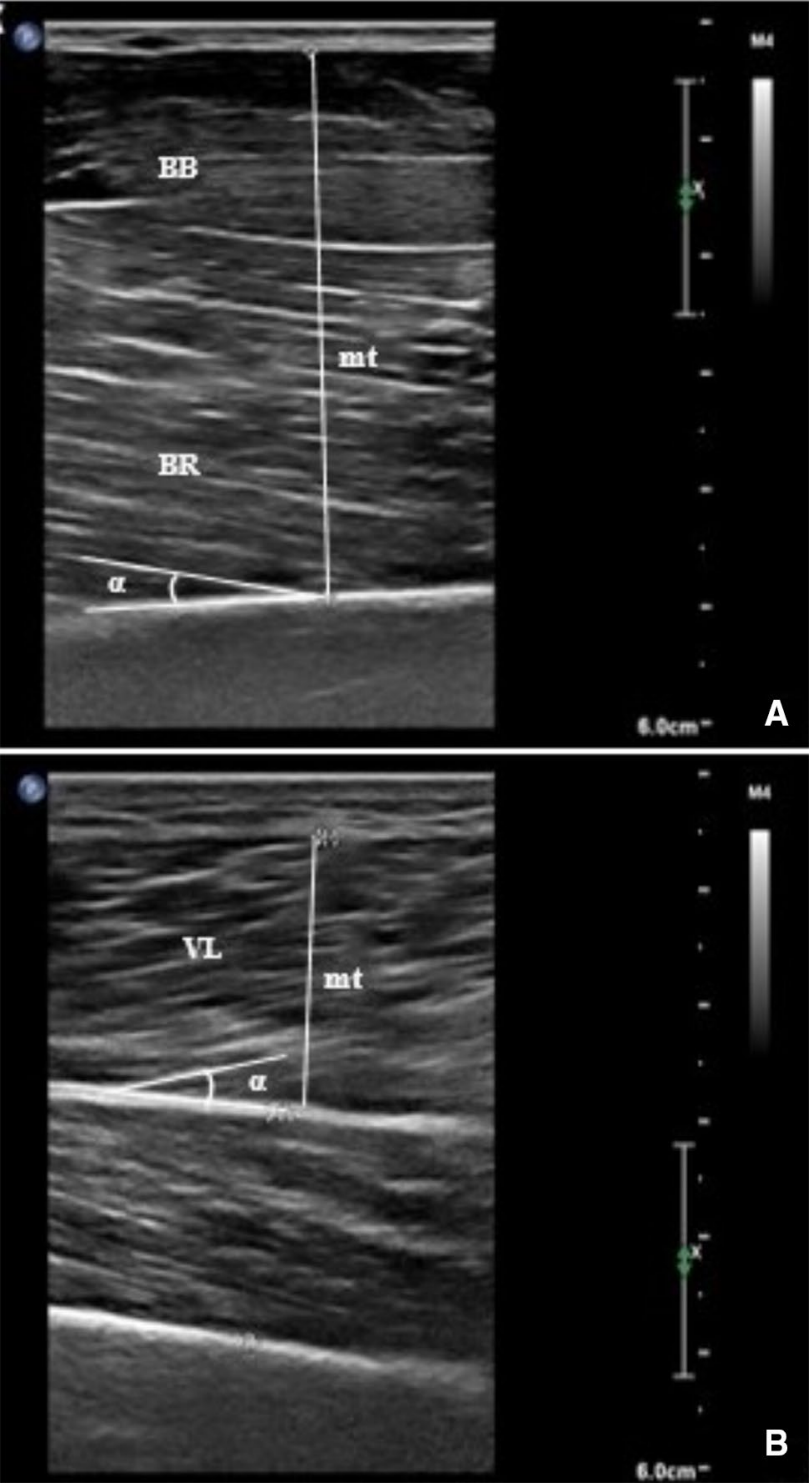
Sagittal ultrasound images: elbow flexors muscle thickness (mt) and pennation angle (*α*) of brachialis (**a**). Muscle thickness (mt) and pennation angle (*α*) of vastus lateralis (**b**)

Muscular thickness was determined as the distance between superficial and deep muscle aponeurosis for the VL, or the superficial aponeurosis of the muscle and muscle-bone boundary for the EF, TB and VM. The pennation angle of the VL was measured by the acute angle between the line of action of the tendon and the line of the muscle fibers. In the case of BR, the angle subtended by the muscle fibers and their bone attachment which is not dependent on joint angle when the muscle is relaxed, was considered (Herbert and Gandevia 1995). Figure 2 shows examples of the ultrasonography images of the site of measurements for the muscle architecture in BR and VL.

For measuring the thickness of the TB, participants stood with their arm alongside their body, in a fully extended position. For the remaining sites, participants were placed in a semi-recumbent and relaxed position with knees fully extended and arms held straight alongside the torso, with a supination position of the lower arms. The measurement sites were accurately located and marked at 60% of the distance from the posterior surfaces of the acromion to the lateral epicondyle of the humerus for TB, and from the coracoid process of the scapula to the medial epicondyle of the humerus for EF. VM and VL, were located and marked at 80% and 60% of the distance between the lateral condyle of the femur and the greater trochanter, respectively (representing the midline on the midsagittal plane).

At each marked site, a 7.5-MHz linear-array transducer together with water-soluble transmission gel (Aquasonic 100 Ultrasound Transmission gel, providing an acoustic coupling during the test without depressing the dermal surface) was placed perpendicular to the skin surface and parallel to the long axis of the muscle. The distortion of tissue due to excessive compression was eliminated by (1) resting the transducer lightly on the skin surface, (2) visually monitoring the image on the ultrasound screen and (3) asking participants to provide verbal feedback on the amount of pressure experienced upon the skin.

Three images of each location were obtained, and the median of the measurements was calculated and used for the analysis, before and after the intervention. To ensure that the location was fully replicated, the position of the probe was recorded onto acetate paper and pre and post-intervention images were compared during the measurements based on identifiable markings (moles and small angiomas) viewed on the skin surface as reference points. This was done to increase the reliability of repeated measures. To avoid osmotic fluid shifts (muscle swelling) which may distort measurements of angle and thickness (Stasinaki et al. 2018), images were obtained at least 48 h after the last training session and prior to the maximal strength tests. The intra-rater reliability of muscle thickness and angle of pennation measurements performed by a single trained investigator on the same scans in a preparatory study was excellent (> 0.99). Therefore, the thickness and the angle of pennation measurements on the four and two, respectively, measured sites and, analyzed at pre- and post-intervention could be confidently compared.

*Countermovement jump (CMJ)* From a standing erect position, the participants descended to a self-selected depth and immediately jumped upwards as high as possible. To exclude the influence of an arm-swing, participants were instructed to keep their hands on their hips (Harman et al. 1990). The CMJ was performed on a Kistler force platform (928B, 3 component force platform; Kistler, Hook, United Kingdom; dimensions: 900 × 600 × 100 mm) with a sampling rate of 2000 Hz. Jump height was calculated from the difference between maximum height of the center of mass (apex) and the last contact of the toe on the ground during the take-off. Test–retest reliability coefficients (ICCs) for the day-to-day reproducibility of the dependent performance measures were recorded at ICCs ≥ 0.90 and the coefficients of variation (CV) ranged from 1.0 to 2.5%.

*Upper body strength* The highest possible weight lifted in one maximal repetition (1RM) for the BP exercise using free weights was determined according to the methodology described by McGuigan (2016). The test–retest intra-class reliability for the two assessed exercises was *R* > 0.93 to < 0.98.

*Upper body mechanical power* This was measured for the BP exercise using 50% of the previously determined 1RM value. Participants were required to perform three maximal velocity repetitions with correct exercise technique. The repetition that produced the maximal average value of the mechanical power (calculated from the accelerative portion of the concentric phase, during which the acceleration of the barbell was ≥ − 9.81 m s^−2^) was selected for the analysis. A recently validated (Laza-Cagigas et al. 2018) portable single optoelectronic infrared camera system (Velowin, Deportec, Spain) with a fixed sampling frequency of 500 Hz was used to track a retroreflective strip placed at the center of the bar during the three BP repetitions. The device was connected to a computer through a USB interface and the proprietary software (Velowin 1.6.314). Numeric and graphical real-time information after each repetition was obtained. All data were filtered using a low pass 10 Hz cut-off filter prior to calculating the displacement of the bar, the movement velocity, the generated force and the produced mechanical power. The test–retest reliability coefficients (ICCs), coefficient of variation (CV) and standard error of measurement (SEM) for the BP mechanical power at 50% were 0.92, 2.0% and 20.10, respectively.

*Measurement of delayed onset muscle soreness (DOMS)* Muscle soreness in anterior and posterior thigh (lower limb) was evaluated at pre- and post-intervention before and after (12-h, 24-h, 48-h) performing a single bout of the EIMS. The EIMS involved ten sets of ten repetitions with 1-minute rest between the sets of a squat exercise using a YoYo-Squat isoinertial flywheel machine (Inertial Power SRL, Santa Fee, Argentina). To cause DOMS, the flywheel device was used to intentionally increase the quadriceps eccentric activation.

Participants were asked to perform a standardized warm-up involving slow squat movements without external overload, to walk and to slowly jog. Thereafter, participants evaluated lower extremity muscle soreness on a visual analogue scale (VAS) of 100 mm ranging from no pain at all (0 mm) to worst possible pain (100 mm) as described elsewhere (Bijur et al. 2001). Following the same procedures and assisted by the same researcher, muscle soreness evaluations at 12-h, 24-h and 48-h were given by all participants,

*Dietary monitoring* Each participant’s baseline diet (3 days, 2 weekdays, and 1 weekend day) was analyzed using Dietplan 7 software (Forestfield Software Ltd, West Sussex, UK). The average relative amount in g kg^−1^ BM^−1^ of proteins, carbohydrates and fat, was as follows: MIC 1.7 ± 0.4, 3.2 ± 1.5, 0.9 ± 0.3; SH 1.6 ± 0.3; 2.9 ± 0.6, 0.8 ± 0.3. The relative daily energy intake was 28.1 ± 5.7 kcal kg^−1^ BM^−1^ and 26.3 ± 5.1 kcal kg^−1^ BM^−1^ for MIC and SH respectively. No between groups significant differences in the macronutrient intake or energy consumption were identified. Participants were instructed to maintain their normal diet throughout the intervention. To avoid potential confounding effects from their diet, participants were instructed not to change their nutritional habits. Importantly, they were asked to report any minimal change regarding food composition and serving-size, or compliance with the reported meals including breakfast, lunch, post-workout food intake and dinner. If any change in diet patterns was reported or identified (i.e. becoming vegetarian, restricting calories, taking nutritional supplements, etc.) participants’ data would have been excluded from the analysis.

*Training protocol and control of intervention compliance* All participants followed the same non-consecutive days resistance training routine (three times per week) for a total of 8 weeks. No other structured physical activities workouts were allowed for the entire intervention period.

Workout sessions were carried out in the late afternoon or early evening. After a standardized warm-up, participants performed a total of three circuits involving one set of the following exercises: (1) parallel squat (2) hang clean (3) bench press (4) upright row (5) double leg dead lift (6) shoulder press (7) alternate lunges with dumbbells (8) push press, and (9) biceps curl. Every set involved ten self-determined maximum repetitions (Steele et al. 2017) using the heaviest possible load and performed with the maximum possible movement velocity. Experienced strength and conditioning coaches monitored all training sessions to ensure participants compliance with the training protocol. When participants were able to perform more than ten repetitions per set, loads were slightly increased (between 2.5 and 5 kg). If less than ten repetitions were completed, a minimum rest period of 15 s was introduced until participants were able to complete the required ten repetitions per set. A ~ 30 s rest period was permitted between exercises. The recovery period between circuits was 2–3 min. All participants completed the total prescribed number of repetitions for each exercise. The average time to complete one workout was 50 min. The resistance-training routine was designed to increase strength and muscle mass of all major muscle groups. A range of ten maximum repetitions using the highest relative load performed with the maximal possible movement velocity was chosen to induce a high level of mechanical and metabolic stress (Denton and Cronin 2006), as well as to favor strength and likely mechanical power improvements (Schoenfeld et al. 2014).

*Intervention* After completing the initial evaluation and in accordance with the randomization, each participant received a microcurrent or sham device and began the intervention. Participants were instructed to wear the microcurrent or sham device for 3-h immediately after the completion of each training session or in the morning during non-training days.

The Arc4Sports (ARC Microtech Ltd, East Sussex, UK) is a rechargeable battery-operated commercially available microcurrent device that sends a pulsating stream of electrons in a relatively low concentration throughout the body (between 2 and 11 pulses per bunch). The device allows the application of a non-invasive protocol and delivers ubiquitous electrical currents that mimic the endogenous electrical energy of the human body. Set by the manufacturer, the output channel utilizes a complex pulsed waveform with a fundamental frequency of 1.0309 kHz, which is given in bursts of varying length and separation. The intensity of the current varies between 50 and 400 μA in a ratio of 2:1 (on:off), using two blocks involving two consecutive cycles of 5 min:2.5 min and 10 min:5min, for a duration of 45 min each cycle (3 h in total). The effect of the microcurrent is to induce a flow of electrons into the tissue.

Both the microcurrent and sham devices were identical in appearance, i.e. size [45 mm (width) × 15 mm (depth) × 105 mm (length)], color and weight (~ 64 g)]. Since the current transmitted from the microcurrent device is insufficient to stimulate sensory nerve fibers, the stimulus was imperceptible and consequently neither participants nor researchers were able to identify participants’ group allocation. One independent researcher, who was not in contact with participants, decoded the devices after completing the analysis of the data.

The same testing procedures were repeated at the end of the intervention. Potential adverse events and compliance with the treatments were evaluated continuously by an individual follow-up of the participants. The researchers controlled compliance with the treatment regularly using instant phone text messages and checking with the participants during regularly weekly interviews. Only participants completing all training session and declaring 100% compliance using the assigned device were considered for the analysis.

### Statistical analysis

A descriptive analysis was performed and subsequently the Kolmogorov–Smirnov and Shapiro–Francia tests were applied to assess normality. Sample characteristics at baseline were compared between groups using an independent-means Student’s *t* test. All pre- and post-intervention data were summarized and reported as mean ± standard deviation unless stated otherwise. Raw changes in all outcome variables were calculated by subtracting pre from post assessment values. Under the assumption that both conditions would promote changes from baseline values due to the common exercise program and that the amount of change would be also dependent on each individual’s baseline performance levels, one-way analysis of covariance (ANCOVA) models were used to compare differences in raw change between groups, using the pre-assessment values as covariates. Confidence intervals (CI) of the adjusted differences were calculated and plotted. Those CIs not crossing zero were considered statistically significant. Additionally, two-tailed one sample Student’s *t*-tests were used to test for a null effect hypothesis. As DOMS were assessed before and at three time points (12-h, 24-h and 48-h) after completing the EIMS, at pre- and post-intervention, a 3-way [2 (conditions: MIC vs. SH) $\times$ 4 (times: pre, post 12-h; post 24-h and post 48-h) $\times$ 2 moments (pre- vs. post intervention)] repeated-measures analysis of variance (ANOVA) was used. Differences over time were compared using Bonferroni-adjusted pairwise comparisons when appropriate. Eta squared ${(\eta }^{2})$ and Cohen’s *d* standardized effect sizes of the adjusted differences between intervention groups were calculated from the ANCOVA or ANOVA F tests, and compared to common benchmarks (Cohen 1988) (small η^2^ = 0.01, *d* = 0.2; moderate η^2^ = 0.06, *d* = 0.5; and large η^2^ = 0.14, *d* = 0.8).

All statistics were performed using the Statistical Package for the Social Sciences (SPSS for Windows, version 20.0; SPSS, Inc., Chicago, IL, USA). Significance level was set to *p* < 0.05.

## Results

### Body composition, muscle architecture and performance

Table 1 describes the mean and standard deviation values along with the observed absolute changes [95% CI] in body composition (BM, fat mass and fat-free mass), muscle thickness (EF, TB, VM, and VL), angle of pennation (BR and VM) and performance (vertical jump, upper body strength and power) for each of the intervention groups.

**Table 1:** Mean (M) ± standard deviation (SD) of the pre and post values and the changes M ± SD [95% CI] of the analyzed variables for the two intervention groups

Variables	Microcurrent (*n* = 9)			Sham (*n* = 9)			Groups comparisons	
	Pre	Post	Changes	Pre	Post	Changes	*p* value	ES
Body mass (kg)	87.9 ± 11.1	88.9 ± 10.9	0.95 ± 1.4 [− 0.11, 2.00]^t^	89.5 ± 10.3	89.8 ± 10.5	0.30 ± 3.2 [− 2.14, 2.74]	0.58	0.28
Fat mass (kg)	15.9 ± 5.6	10.93 ± 15.7	− 0.16 ± 1.6 [− 1.39, 1.08]	15.9 ± 7.9	15.5 ± 8.5	− 0.36 ± 1.9 [− 1.85, 1.14]	0.81	0.12
Fat-free mass (kg)	72.1 ± 10.6	73.2 ± 10.9	1.0 ± 1.4 [− 0.09, 2.09]^t^	73.5 ± 6.2	74.3 ± 6.5	0.76 ± 1.7 [− 0.55, 2.07)	0.75	0.16
Fat mass (%)	18.0 ± 5.7	17.8 ± 5.1	− 0.23 ± 1.9 [− 167, 1.22]	17.3 ± 6.9	16.8 ± 7.1	− 0.49 ± 1.7 [− 1.77, 0.80]	0.76	0.15
Fat-free mass (%)	81.8 ± 5.6	82.4 ± 5.5	0.58 ± 0.6 [− 1.21, 2.36]	82.6 ± 6.7	83.2 ± 7.1	0.68 ± 0.7 [− 0.51, 1.86]	0.92	0.05
Elbow flexors thickness (mm)	39.2 ± 3.0	42.1 ± 3.0	2.9 ± 1.4 [1.8, 3.9]**	38.4 ± 6.2	41.7 ± 5.8	3.0 ± 2.4 ± [1.2, 4.9]**	0.89	0.07
Triceps brachii thickness (mm)	29.3 ± 5.6	33.6 ± 6.3	4.3 ± 2.8 [2.2, 6.5]**	28.8 ± 4.9	31.4 ± 7.5	2.7 ± 2.6 [0.6, 4.7]*	0.22	0.64
Vastus medialis thickness (mm)	35.8 ± 5.5	37.2 ± 5.3	1.5 ± 1.5 [0.3, 2.6]*	35.0 ± 2.3	36.1 ± 2.7	0.9 ± 0.8 [0.2, 1.5]*	0.34	0.49
Vastus lateralis thickness (mm)	24.4 ± 8.6	31.2 ± 12.0	6.8 ± 8.0 [0.7, 12.9]*	27.0 ± 9.9	30.2 ± 11.1	3.2 ± 1.8 [1.8, 4.6]**	0.20	0.66
Brachialis, pennation angle (degrees)	12.4 ± 2.93	14.34 ± 1.33	1.93 ± 1.5 [0.77, 3.09]**	12.8 ± 2.1	13.5 ± 2.2	0.73 ± 0.6 [0.30, 1.16]**	0.04	1.22
Vastus lateralis, pennation angle (°)	14.1 ± 3.42	17.0 ± 3.83	2.90 ± 0.9 [2.17, 3.63]**	16.5 ± 5.5	18.5 ± 5.6	1.90 ± 1.2 [0.90, 2.82]**	0.06	0.99
Vertical jump height (m)	0.28 ± 0.03	0.31 ± 0.05	0.03 ± 0.03 [0.01, 0.05]**	0.27 ± 0.05	0.29 ± 0.06	0.02 ± 0.03 [− 0.01, 0.05]^t^	0.61	0.26
1RM bench press (kg)	100.6 ± 21.7	109.3 ± 23.1	8.7 ± 4.7 [5.15, 12.34]**	96.7 ± 19.4	103.1 ± 16.9	6.4 ± 4.3 [3.0, 9.7]**	0.28	0.55
Mechanical power at 50% 1RM in bench press (watts)	421.4 ± 19.2	557.6 ± 11.4	134 ± 92 [63, 205]**	409.3 ± 16.7	498.2 ± 10.6	79 ± 94 [7, 151]*	0.23	0.26

*p < 0.05, ^**^p < 0.01, ^t^*p* < 0.10 respect to baseline levels; ES is the standardized effect size presented as Cohen’s *d*

**Fig. 3:**
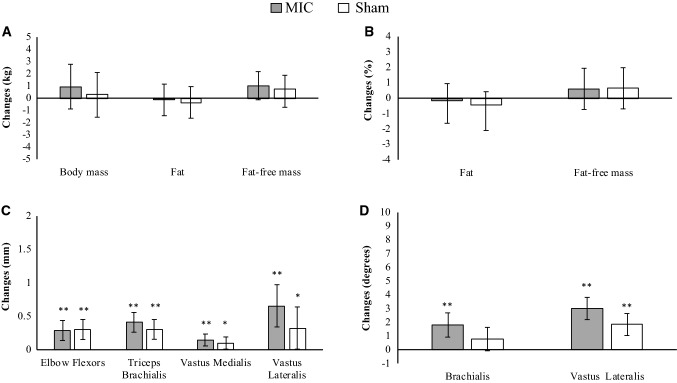
Estimated marginal means and 95% confidence intervals of adjusted changes in body composition (**a**, **b**), muscle thickness (**c**) and the angle of pennation (**d**). Analysis of covariance (ANCOVA) model was used to compare differences in raw change between groups, using the pre-assessment values as covariates. *p < 0.05; **p < 0.01 from the baseline values. *MIC* microcurrent treatment group, *SHAM* sham treatment group

No significant differences were observed at pre-intervention in any of the analyzed variables. Both groups, MIC and SH showed no significant absolute changes in any of the analyzed body composition variables. Nonetheless, it is worth highlighting that the MIC group showed large effect sizes of the absolute changes measured for both BM (*p* = 0.073, *d* = 1.45) and fat-free mass (*p* = 0.069, *d* = 1.48). Indeed, when the adjusted values are considered a moderate effect size (*p* = 0.071, *d* = 0.45) to increase fat-free mass by the MIC group is confirmed (Fig. 3a).

Both groups, MIC and SH produced significant absolute (Table 1) and adjusted (Fig. 3c) increases in the muscle thickness for the four analyzed muscles. However, it is worth noting that when the adjusted values are considered, compared to SH, the MIC group elicited larger effects sizes (*d* = 0.82 vs. *d* = 0.56 and *d* = 1.05 vs. *d* = 0.52) for the VM and VL thickness, respectively.

Both MIC and SH showed absolute significant increase of the angle of pennation measured in both VL and BR (Table 1). Nonetheless, when adjusting by the pre-intervention values, the observed differences were confirmed for the VL in both MIC and SH, while only MIC increased the pennation angle in BR. Furthermore, main significant differences between groups were determined for the angle of pennation at the VL (*p* = 0.045; *d* = 1.10; Fig. 3d), while a large effect size (*p* = 0.094, *d* = 0.90) between the changes measured in the pennation angle of the BR was determined between groups (Fig. 3d).

**Fig. 4:**
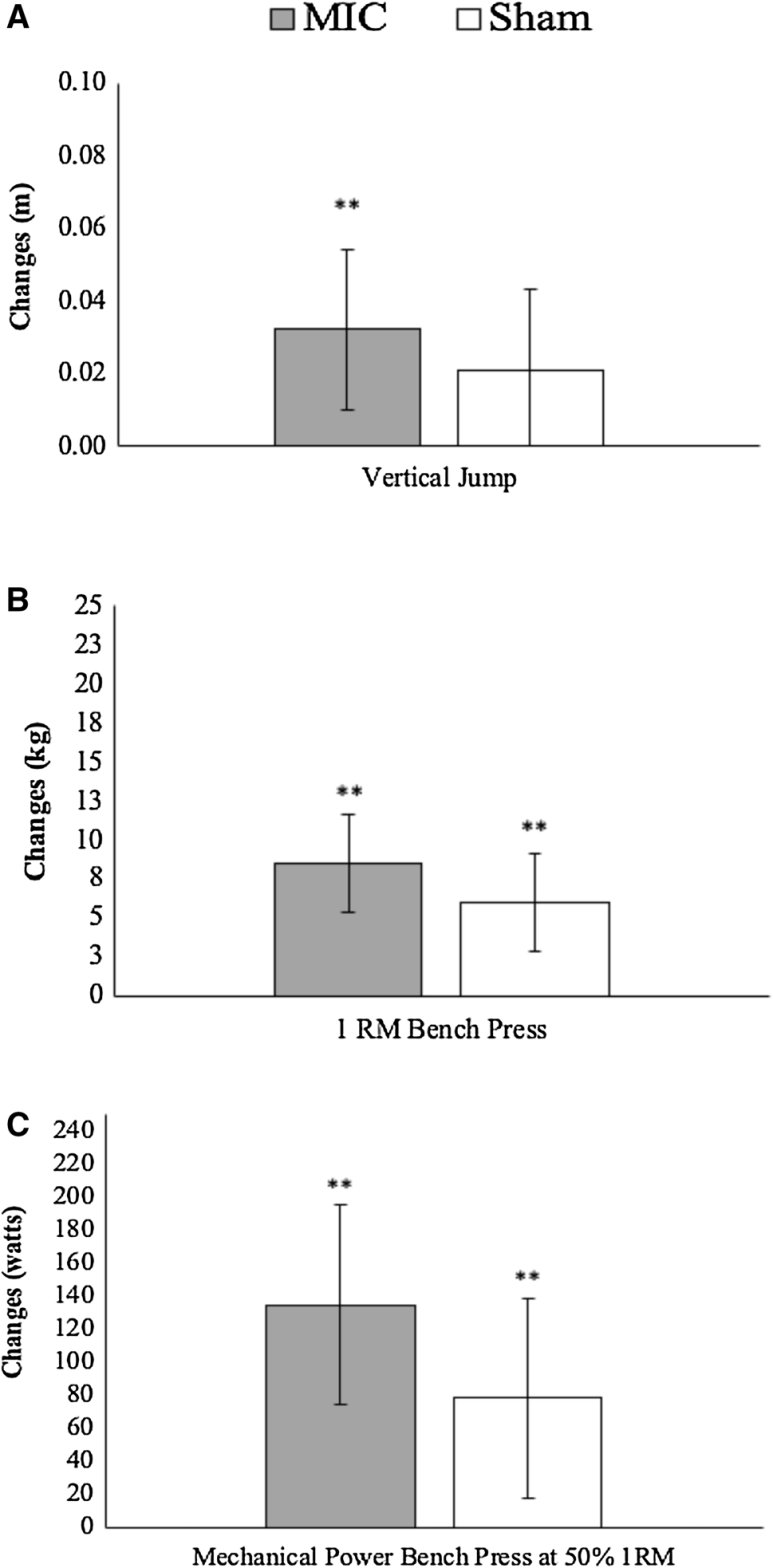
Estimated marginal means and 95% confidence intervals of adjusted changes in vertical jump height (**a**), 1RM bench press (**b**) and mechanical power in bench press at 50% of 1RM (**c**). Analysis of covariance (ANCOVA) model was used to compare differences in raw change between groups, using the pre-assessment values as covariates. *p < 0.05; **p < 0.01 from the baseline values. *1RM* 1 repetition maximum, *MIC* microcurrent treatment group, *SHAM* sham treatment group

Regarding performance, both groups improved the 1RM load and the mechanical power using 50% of 1RM in the BP exercise (Table 1 and Fig. 4b, c). However, only MIC improved vertical jump height while a non-significant (*p* = 0.052) with a moderate effect size (*d* = 0.50) improvement was identified in SH (Fig. 4a). No between-group differences were determined at post-intervention.

### Delayed muscle soreness (DOMS)

A main interaction effect moment × time × group [*F*(3, 16) = 5.34, *p* = 0.003 = ${\pi }^{2}$0.25] was determined.

**Fig. 5:**
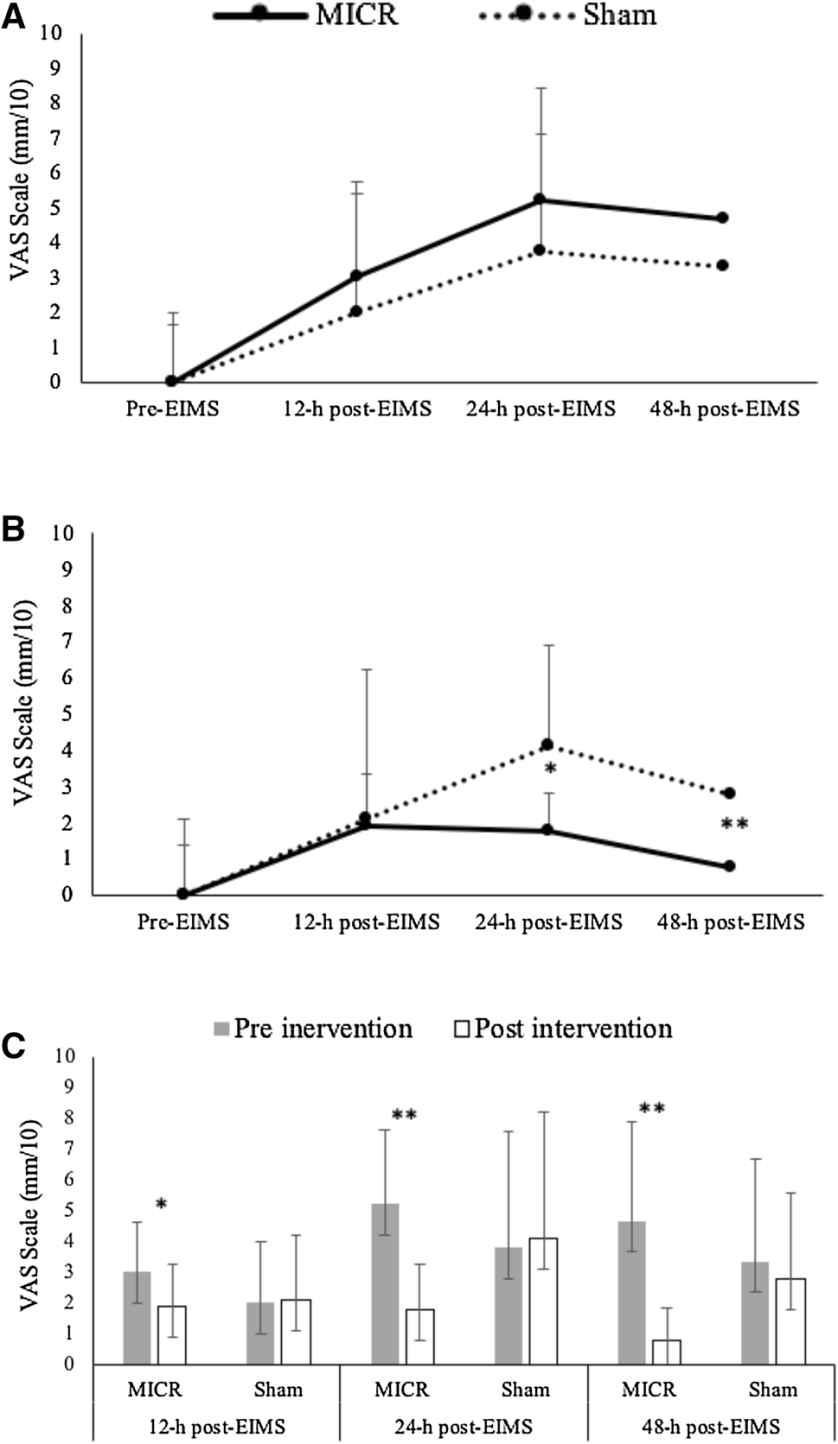
Mean and standard deviation of the delayed muscle soreness measured from the visual analogue (VAS) scale at pre intervention (**a**), post-intervention (**b**) and between pre- and post-intervention classified by group and post-EIMS time points (**c**). *p < 0.05; **p < 0.01 between groups (**a**, **b**); from pre to post (**c**). *MIC* microcurrent treatment group, *SHAM* sham treatment group, *EIMS* exercise-induced muscle soreness protocol

At pre-intervention, significant increases (*p* < 0.05) from the pre-EIMS values were observed at the three post-EIMS time points (12, 24, and 48-h) for both intervention groups. In addition, the level of DOMS expressed at 24-h and 48-h was similar between the groups (*p* > 0.39) and significantly higher (*p* < 0.05) than the DOMS expressed at 12-h in both groups (Fig. 5a). No significant difference between groups was observed at any time for the pre-intervention assessment.

At post-intervention, the SH group showed a very similar pattern of response with respect to the level observed at pre-intervention. Significantly higher DOMS (*p* < 0.01) were measured at 12-h, 24-h and 48-h in respect to baseline levels. Nonetheless, after the intervention, the values measured at 24-h were similar (*p* = 0.27) to the values determined at 12-h but, respectively, higher to those measured at 48-h (*p* = 0.01) (Fig. 5b). Conversely the MIC group produced a very different response pattern. A significant increase (*p* < 0.05) of DOMS, respectively, to baseline was observed at 12-h (*p* = 0.01) and 24-h (*p* = 0.012) but not at 48-h (*p* = 0.12). Furthermore, the level of DOMS measured at 12-h and 24-h were similar (*p* = 0.86) but still higher (*p* < 0.05) than those determined at 48-h (Fig. 5b).

When the values determined before and after intervention were compared (Fig. 5c), only the MIC group showed significant reductions of DOMS at the three post-EIMS time points (12-h; 24-h and 48-h). No significant differences were determined for the sham group.

## Discussion

Results of the present study suggest that wearing a microcurrent device with an intensity varying between 50 and 400 μA along with a fundamental frequency of ~ 1 kHz, for a total of 3 h after workouts or during the morning in non-training days, produced no additional statistical significant benefits on body composition, including the optimization of the training-induced hypertrophy, and performance over an 8-week intervention period. However, beneficial effects were observed on muscle architecture by increasing the pennation angle of VL and possibly that of BR beyond the changes induced by the exercise intervention alone. Notwithstanding, in line with previous investigations (Lambert et al. 2002; Curtis et al. 2010; Kwon et al. 2017), the most relevant effect of using a microcurrent treatment parallel to resistance training is the reduction of DOMS perception determined after a very hard concentric-eccentric EIMS. Based on the observed results we have to reject our hypothesis that supports the additive effect of a microcurrent treatment to maximize resistance training outcomes on body composition, hypertrophy and performance. Conversely, our hypothesis can be confirmed with regards to the effect of microcurrent eliciting changes in the angle of pennation and with regards to the attenuation of DOMS measured over a period of 12-h to 48-h.

The food analysis revealed similar amounts of macronutrients and caloric intake for both groups. Regardless of group, the daily protein consumption for all participants was between 1.2 and 2 g/kg of BM. This figure is within the accepted range to support muscle mass accretion in resistance-trained individuals (Jager et al. 2017). It also approximates the recommended value of 1.6 g/kg/day to support lean mass accretion by resistance training interventions (Morton et al. 2018). In the context of the present study, no limitations associated with sub-optimal nutrition should have consequently affected the observed results.

Although no statistically significant differences favoring body composition outcomes on the MIC were observed at post-intervention, the larger effect sizes in terms of increasing fat-free mass and enlarging both, VM and VL thickness suggest a potential additive effect of the applied microcurrent treatment, contributing to optimize the hypertrophic response that was more noticeable in lower body musculature. The length of the training program, i.e. 8 weeks, using relatively well-trained participants, although enough to elicit training adaption, can also be suggested as insufficient duration to create an appropriate summative microcurrent-induced hypertrophic effect, consequently precluding the attainment of statistically significant differences between interventions.

The increase of the pennation angle determined for both intervention groups, MIC and SH can be considered as a normally expected outcome resulting from strength training programs aimed to increase muscle mass and successfully enlarging cross sectional areas (Aagaard et al. 2001). In pennate muscles such as VL, a steeper pennation angle of the muscular fibers provides a larger physiological fiber area for a given muscle volume and therefore more potentially activated actin-myosin cross bridges, which results in greater strength and force generation (Suetta et al. 2008). Similarly, a greater angle of pennation related to the deep aponeuroses of a typically parallel fiber muscle as BR (de Boer et al. 2008) can be indicative of an increased capacity of force production. From this point of view, it would be reasonable to expect that the larger angle of pennation produced by the MIC compared to SH for VL also impacted on exercise performance improvements. In support of the previous rationale, although no difference between groups was observed at the end of the intervention period, only MIC produced significantly increased vertical jump heights, while a non-significant improvement was determined for the SH condition (Fig. 4a). On the other hand, both groups similarly improved BP 1RM and mechanical power values. Despite no pennation angle of synergistic muscles involved in the BP exercise, such as triceps brachialis was measured and, no exercise demanding a meaningful action of the BR was used for assessing changes in performance, it seems that the used microcurrent protocol was slightly more effective on maximizing adaptations in lower body musculature. Furthermore, the applied training routine imposed a higher volume of work on VL by active recruitment during four exercises (parallel squat, hang clean; and alternate lunges) whilst the BR was mainly activated in only one exercise (biceps curl). These differences on the training overload may have impacted on the observed results.

Pennation is a strategy to pack greater numbers of contractile elements along the aponeurosis and tendon (Narici 1999). The observed enlargement of the muscular thickness along with the increased pennation angle can be considered indicative of added sarcomeres in parallel with a physiologically adaptive outcome that favors the capacity to generate force (Kawakami et al. 2006). Although the training intervention seems to be the main mechanical stimulus for eliciting these aforementioned adaptions, the overall larger effect sizes favoring MIC vs. SH to increase muscle thickness along with the higher pennation angles measured in MIC allow us to suggest that combining microcurrent with resistance training could represent an appropriate method to maximize training outcomes in resistance-trained individuals. The mechanisms associated with this training-induced effect optimization are still unclear but they can be linked to an increased muscle membrane sensitivity in response to mechanical stimulus favoring a more efficient upregulation of muscle protein synthesis and recovery after each singular workout (Ohno et al. 2013; Fujiya et al. 2015). In fact, the application of microcurrent in mouse cell culture upregulated the expression of MM creatine kinase, Caveolin-3 and tripartite motifcontaining 72, which are proteins related to muscle growth and remodeling (Ohno et al. 2019). Additionally, a transient increase in the relative expression of protein kinase B (p-Akt), which supports the promotion of muscle anabolism and the reduction of protein degradation via mTORC1 (Morley 2016) was also reported (Ohno et al. 2019). In this context Kwon et al. (2017) observed beneficial effects of a short-term 40 min microcurrent treatment to improve handgrip strength, lower body endurance and muscular efficiency in elderly individuals. These authors suggested that, as detected in animal models (Ohno et al. 2013; Fujiya et al. 2015), microcurrent can help in restoring or regenerating damaged muscles by local stem cell activation. Indeed, as observed in the present investigation, the most often reported effect of microcurrent is the reduction in the perception of muscle soreness (Lambert et al. 2002; Curtis et al. 2010). The level of muscle soreness is also the most common assessed marker of exercise-induced muscle damage (Warren et al. 1999), representing a complex interaction of disruption of muscle structure, alteration of the calcium (Ca^2+^) homeostasis and sensitization of nociceptors from inflammatory cell infiltrates (Hyldahl and Hubal 2014). Excessive accumulation of intracellular Ca^2+^ can alter membrane integrity, which gradually induces morphological and functional changes in the skeletal muscle contractile structure (Kwon et al. 2017). The post-exercise application of microcurrent could have therefore supported the maintenance of intracellular Ca^2+^ homeostasis in potentially disrupted muscles after performing exhaustive exercise using a strong eccentric component as the EIMS performed by our participants (Lambert et al. 2002). Consequently, the reduced perception of DOMS experienced by the MIC group could be associated with a more efficient capacity of the muscles to tolerate and adapt to a hard exercise bout. This is of relevance in sports where muscle damage can impact upon subsequent workouts and competitions (Owens et al. 2018). Some of the proposed mechanisms of microcurrent-induced attenuation of DOMS is the effect of hastening muscle protein synthesis, in addition to satellite cell response and proliferation, which are necessary in improving post-workout muscle regeneration (Fujiya et al. 2015; Hiroshige et al. 2018).

Our study is not without limitations: the intervention period lasted only 8 weeks and although this period can be considered sufficient to elicit measurable changes on the analyzed dependent variables, it is possible that results between groups could have diverged with a longer implemented intervention protocol. No muscle fiber composition analysis was conducted. Although a heavy resistance training routine, like the one used by our participants, tends to produce hypertrophy of type I and II fibers; type II fibers enlarge proportionately more than type I fibers (Kraemer et al. 1996). It could also be possible that participants with a higher proportion of fast twitch fibers distributed towards the periphery produced a larger hypertrophy response, which was underestimated by measuring the thickness at the middle region of the muscles as in the analyzed muscles (elbow flexors, triceps brachii extensors and quadriceps) type II fibers predominate around the periphery of the fascicles (Manta et al. 1996). Furthermore, diet was not fully controlled but participants were instructed to maintain their habitual diet habit and report any significant change in the feeding behavior. Providing a prepared and prepacked diet to participants during the study would have offered an ideal scenario to standardize and control the influence of diet on the present results. Furthermore, as only resistance-trained males were assessed in the present study, further studies involving females are required.

In conclusion, although no significant differences between treatment groups were observed after 8 weeks of resistance training with respect to improvements in body composition, hypertrophy and performance outcomes, a 3-h daily application of microcurrent-maximized muscular architectural changes and attenuated the perception of DOMS in resistance-trained men.

## Abbreviations

1RM: One maximal repetition
ANCOVA: Analysis of covariance
ANOVA: Analysis of variance
ATP: Adenosine triphosphate
BM: Body mass
BP: Bench press
BR: Brachialis
CI: Confidence intervals
CMJ: Countermovement jump
CV: Coefficient of variation
DOMS: Delayed onset muscle soreness
EF: Elbow flexors
EIMS: Exercise-induced muscle soreness protocol
FFM: Fat-free mass
FM: Fat mass
ICCs: Test–retest reliability coefficients
kcal: Kilocalories
kg: Kilogram
MIC: Intervention group using the microcurrent device
p-Akt: Protein kinase B
SEM: Standard error of measurement
SH: Intervention group using the sham device
TB: Triceps brachii
VAS: Visual analogue scale
VL: Vastus lateralis
VM: Vastus medialis
