# Correction to: Effectiveness of combining microcurrent with resistance training in trained males

**DOI:** 10.1007/s00421-019-04294-4

**Published:** 2020-01-09

**Authors:** Fernando Naclerio, Marcos Seijo, Bettina Karsten, George Brooker, Leandro Carbone, Jack Thirkell, Eneko Larumbe-Zabala

**Affiliations:** 1 Department of Life and Sport Science, University of Greenwich, Avery Hill Campus, Sparrows Farm, Avery Hill Road, Eltham, SE9 2BT UK; 2 Department of Exercise and Sport Science, Lunex International University of Health, Exercise and Sports, Differdange, Luxemburg; 3 Department of Biological Sciences, Royal Holloway, University of London, London, UK; 4 Clinical Research Institute, Texas Tech University Health Sciences Center, Lubbock, TX USA

## Correction to: European Journal of Applied Physiology (2019) 119:2641–2653 10.1007/s00421-019-04243-1

The original version of this article unfortunately contained a mistake. The word post exercise is written twice at the end of the first line of the “Introduction section” of the abstract.

The Introduction should read as:

Microcurrent has been used to promote tissue healing after injury or to hasten muscle remodeling post exercise.

